# The role of photon recycling in perovskite light-emitting diodes

**DOI:** 10.1038/s41467-020-14401-1

**Published:** 2020-01-30

**Authors:** Changsoon Cho, Baodan Zhao, Gregory D. Tainter, Jung-Yong Lee, Richard H. Friend, Dawei Di, Felix Deschler, Neil C. Greenham

**Affiliations:** 1 Cavendish Laboratory, Department of Physics, University of Cambridge, J.J. Thomson Avenue, Cambridge, CB3 0HE UK; 2 School of Electrical Engineering, Korea Advanced Institute of Science and Technology (KAIST), Daejeon, 34141 Republic of Korea; 3 State Key Laboratory of Morden Optical Instrumentation, College of Optical Science and Engineering, International Research Center for Advanced Photonics, Zhejiang University, Hangzhou, 310027 China; 4 Present Address: Dresden Integrated Center for Applied Physics and Photonic Materials (IAPP), Technische Universität Dresden, Dresden, 01187 Germany; 5 Present Address: Walter Schottky Institut, Technische Universität München, Garching, D-85748 Germany

**Keywords:** Materials for devices, Lasers, LEDs and light sources

## Abstract

Perovskite light-emitting diodes have recently broken the 20% barrier for external quantum efficiency. These values cannot be explained with classical models for optical outcoupling. Here, we analyse the role of photon recycling (PR) in assisting light extraction from perovskite light-emitting diodes. Spatially-resolved photoluminescence and electroluminescence measurements combined with optical modelling show that repetitive re-absorption and re-emission of photons trapped in substrate and waveguide modes significantly enhance light extraction when the radiation efficiency is sufficiently high. In this manner, PR can contribute more than 70% to the overall emission, in agreement with recently-reported high efficiencies. While an outcoupling efficiency of 100% is theoretically possible with PR, parasitic absorption losses due to absorption from the electrodes are shown to limit practical efficiencies in current device architectures. To overcome the present limits, we propose a future configuration with a reduced injection electrode area to drive the efficiency toward 100%.

## Abstract (web summary)

Perovskite light-emitting diodes have shown unexpected high external quantum efficiency of 20%, breaking the ray-optics limit. Here Cho et al. reveal that photon recycling is responsible for the enhancement and propose photonic structures to further improve the device efficiency.

## Introduction

Perovskite light-emitting diodes (PeLEDs) have recently shown great advances in performance and have reached external quantum efficiencies (EQEs) of up to 22%^1–6^, comparable to those of state-of-the-art organic LEDs (OLEDs)^7–9^. It is notable that the reported EQEs are significantly above the ray-optics limit^10,11^ of 1/2*n*^2^ (corresponding to between 7 and 13%), as typical perovskites have relatively large refractive indices, *n*, between 2 and 2.6^2,12–15^ compared with organic emitters with *n* < 1.8^7,16^. Although there have been many reports of OLEDs overcoming this limit by optimising microcavity effects with thin emissive layers^7,8,17^, it is surprising that PeLEDs have achieved efficiencies of almost twice the limit even with thick emissive layers (~ 200 nm)^2,4^ in which the positions and orientations of emissive dipoles are difficult to control and the corresponding wave-optical effects are diluted. This discrepancy raises three questions: (i) how do PeLEDs achieve high EQEs overcoming their low ray-optical limit; (ii) what internal quantum efficiencies (IQEs) and light extraction efficiencies (LEEs) are being achieved in current PeLED devices; and (iii) what is the upper limit of PeLED efficiency and how might it be obtained?

Perovskite semiconductors have relatively small Stokes shifts, which lead to significant levels of re-absorption of emitted photons^3–5,18,19^. Although re-absorption has been typically considered as a loss mechanism in LEDs^20,21^, with sufficient luminescence efficiency, the process of photon recycling (PR) can assist with optical outcoupling by randomising the direction of photon propagation and redirecting photons from trapped to outcoupled modes^22^. PR has been previously reported to enhance the externally outcoupled photoluminescence (PL) of perovskite films and to improve the voltage characteristics of photovoltaics (PVs)^22–30^. Although PR gives PeLEDs the potential to achieve 100% EQE theoretically, its practical contribution to the device efficiency is still under debate^22–28^.

In the present study, we aim to verify and quantify the contribution of PR in PeLEDs and to suggest design principles to maximise the benefits from PR. Using (PEA)_2_Cs_n−1_Pb_n_Br_3n+1_ with luminescence peaking at 520 nm, where PEA refers to phenylethylammonium, we measure spatially resolved PL and electroluminescence (EL). By detecting recycled photons at large distances from the excitation, we find that PR can significantly increase extraction of photons from trapped modes. Moreover, our modelling approach allows quantitative analysis of the LED efficiency. In state-of-the-art PeLEDs having EQE > 20%, PR is calculated to be able to contribute > 70% of light emission (i.e., an absolute EQE increase of > 14%). Although the recently reported EQEs are already close to calculated limits for planar architectures, we propose several photonic structures maximising the PR effect, which could further improve PeLED performance.

## Results

### Experimental investigation of PR in perovskites

**Fig. 1:**
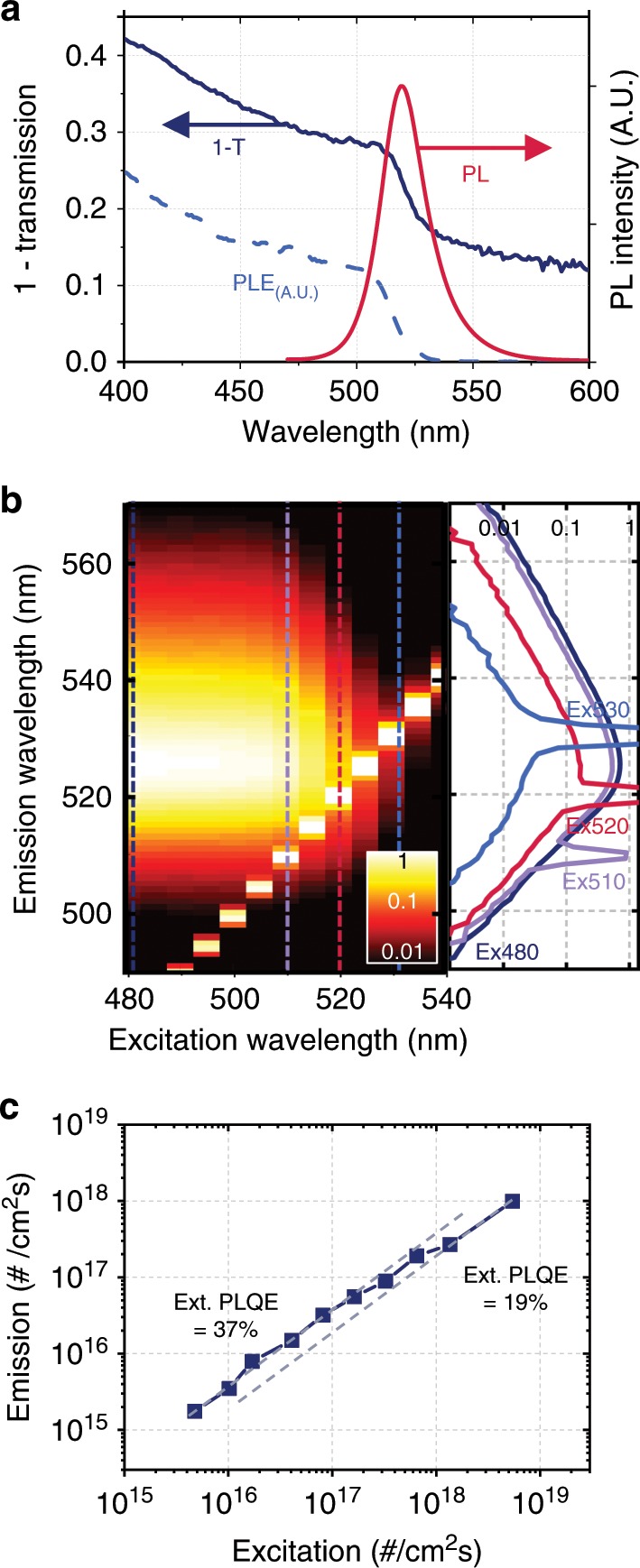
Optical characterisation of perovskite film. **a** Measured extinction (= 1- *transmission*), PL and PLE spectra of a 50 nm-thick perovskite film. **b** Measured normalised PL spectra as a function of excitation wavelength. (left: excitation-scanned colour map. Right: spectra at excitation wavelengths of 480, 510, 520 and 530 nm.) The sharp peaks seen in the emission spectra are owing to detection of photons from the excitation source; for 530 nm excitation the spectral tail of the detected excitation causes some distortion of the overall spectrum. **c** Flux of emitted photons as a function of the flux of absorbed 405 nm photons, with lines indicating PLQEs of 37% and 19%.

In this study, we used films of the 2D–3D mixture PEA_2_Cs_n–1_Pb_n_Br_3n+1_ perovskite having high PL efficiency and a smooth surface (roughness of ~ 0.55 nm (Supplementary Fig. 1)). Figure 1a shows the optical extinction and PL spectra of investigated samples. Although the tail region of the extinction spectrum at wavelengths above 530 nm comes from Fresnel reflection at film interfaces, the absorption seen below 530 nm (Supplementary Fig. 6c) overlaps with the emission spectrum (Stokes shift of ~ 10 nm), which implies significant re-absorption. We measured the PL excitation (PLE) spectrum, representing the relative PL intensity as a function of excitation wavelength. The shape of PLE spectrum follows the absorption characteristics (Fig. 1a) and the peak PL wavelength is almost constant as the excitation wavelength is scanned across the spectral region of absorption (Fig. 1b). This implies that the PL efficiency and spectrum do not depend on the wavelength of absorbed photons, and are sustained even in the region of re-absorption including wavelengths slightly longer than the peak wavelength^31^. As shown in Fig. 1c, in contrast to 3D perovskites used in previous studies for PR^24,25,27^, the external PL quantum efficiency (PLQE) of the perovskite is higher at low excitation fluxes (37%) than at higher excitation fluxes (19%) (probably owing to increased Auger recombination^25,28^), allowing more precise investigation of the effect of PR at distances away from the original excitation.

**Fig. 2:**
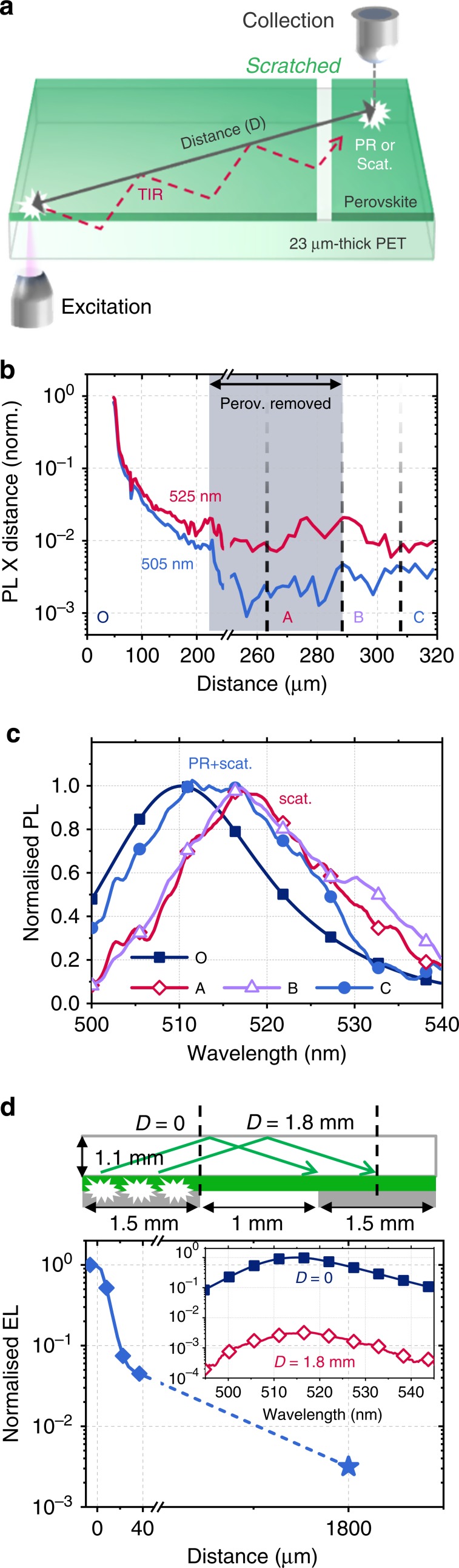
Laterally measured PL and EL of perovskite. **a** Schematic representation of spatially resolved PL measurements with separate microscope objectives for excitation and collection. Trapped photons become detectable by either PR or scattering. **b** Laterally measured PL (normalised) as a function of distance from the point-of-excitation at wavelengths of 505 (± 5) nm and 525 (± 5) nm for a 2D–3D mixture PEA_2_Cs_n–1_Pb_n_Br_3n+1_ perovskite film with a gap between 230 μm and 290 μm. The measured PL intensity was multiplied by distance to compensate for the radial spread of light intensity, to give an indication of photon losses as a function of distance. **c** Measured PL spectra at distances of 0 μm (O, initial excitation), 265 μm (A, where perovskite was removed), 290 μm (B, at the edge of the gap) and 310 μm (C, on perovskite). **d** Normalised spatial EL for a full device, where voltage (4 V) is applied to the pixel covering from  −1500 μm to 0 μm. (inset: the measured EL spectra in A.U. at 0 μm and 1800 μm). The schematic shows possible ray paths in the substrate.

Figure 2a presents a schematic of the spatially resolved PL measurement setup used, in which the lateral distance between the two objectives for 405 nm laser excitation and PL collection are varied. The 80 nm-thick perovskite film was coated on a 23 μm-thick polyethylene terephthalate (PET, Hostaphan RN 23) substrate. The radiated photons not contained in the escape cone are trapped by total internal reflection at the interfaces and diffuse radially. Since the sweep distance (300 μm) is orders of magnitude larger than both the re-absorption distance (1/*α*_520nm_ = 1.3 μm in our perovskite) and the typical charge carrier diffusion lengths (on the order of 1 μm) of perovskites^32–34^, the signal collected at such large distances is expected to arise mostly from re-absorbed photons in the substrate mode rather than those in the waveguide mode or from diffused charge carriers.

As shown in Fig. 2b, we observe a small amount of light outcoupled at large distances from the point of excitation for both 505 nm and 525 nm. Although both scattering and PR can make trapped photons detectable at long distances^24^, their contributions can be distinguished by their different spectral characteristics: for a sufficiently narrow PL spectrum, the scattering length does not change significantly for emitted wavelengths and the scattered spectrum represents the spectrum of trapped photons at the relevant position. As trapped photons with shorter wavelengths have higher probability to undergo re-absorption, the trapped light and its scattering signal tend to be more red-shifted along the distance of propagation, whereas the signal from PR always resembles the original PL spectrum, as observed in Fig. 1b. In Fig. 2b, the signals at 505 nm and 525 nm show slightly different distance dependence up to 200 μm (i.e., spectral variation), indicating the existence of this scattering effect.

To quantify the contribution of PR, we created a gap in the perovskite film by mechanically removing the region at distances between 230 μm and 290 μm. Then, as shown in Fig. 2c, the signal A observed in the perovskite-free region shows a purely scattered signal, with a peak that is 10 nm red-shifted from the original signal O at 0 μm. On the other hand, the signal C in the perovskite-covered region after the gap shows a relatively broad spectrum containing both PR and scattered light. PR makes up 55% of the total intensity (Supplementary Fig. 3). Therefore, although significant light scattering is occurring (mostly in the Hostaphan RN 23 PET substrate, known to have a haze of 7%), the recovery of the original PL spectrum at C can be regarded as clear experimental evidence of PR occurring in the perovskite material under investigation. Interestingly, at the far edge (B) of the gap an additional peak appears at ~ 530 nm, not seen in A and C. This arises from scattered outcoupling of photons trapped in the perovskite waveguide mode (Supplementary Fig. 4 and Supplementary Note 2). When photons are recycled (region C), some of them are trapped inside the perovskite thin-film and their spectrum is rapidly red-shifted owing to the short re-absorption length of perovskite. They then are outcoupled at the edge, resulting in the 530 nm peak.

To study the effect of PR in a more practical setting, we measured the spatially resolved EL of a full PeLED device with a structure of glass/indium tin oxide (ITO, 150 nm)/poly(9-vinylcarbazole) (PVK, 10 nm)/perovskite (50 nm)/2,2’,2”-(1,3,5-benzinetriyl)-tris(1-phenyl-1-H-benzimidazole) (TPBi, 50 nm)/LiF (1 nm)/Al, of which the measured maximum EQE was 12.5% at 2.6 V. When a voltage (4 V) is applied to a pixel, as shown in Fig. 2d, light output can be observed even at the centre of the next pixel, which is 1.8 mm away from the edge of the original pixel, with similar spectral characteristics. We attribute this observation to the recycling of photons that have propagated in the substrate mode. The radiation from non-excited film areas raises a practical measurement issue that overall outcoupling efficiency varies depending on the device geometry, including the lateral size and the thickness of the substrate (Supplementary Fig. 5 and Supplementary Note 3).

The outcoupling efficiency of PeLEDs can be calculated by integrating recursive PR processes^24,25,35^. By assuming that the ratios of direct light outcoupling (LEE_0_), perovskite re-absorption (*A*_act_) and parasitic absorption loss (*A*_para_) (LEE_0_ + *A*_act_ + *A*_para_ = 1) are consistent along the recursion, the EQE becomes:

1 $$ EQE_{PR} =	 \, IQE \times LEE_0 \times (1 + {\it{A}}_{{\mathrm{act}}}{\it{ \times \eta }}_{{\mathrm{rad}}}{\it{ + }}{\mathrm{(}}{\it{A}}_{{\mathrm{act}}}{\mathrm{ \times }}{\eta }_{{\mathrm{rad}}}{\mathrm{)}}^{\mathrm{2}}{\it{ + }} \ldots )\\ =	 \, {\eta} _{{\mathrm{inj}}}{\mathrm{ \times }}{\eta} _{{\mathrm{rad}}} \times {\mathrm{LEE}}_0/{\mathrm{ }}(1 - A_{{\mathrm{act}}} \times \eta _{{\mathrm{rad}}})\\ =	 \, {\eta} _{{\mathrm{inj}}} \times {\mathrm{LEE}}_0\;/\;\left( {{\mathrm{LEE}}_0 + A_{{\mathrm{para}}} + \left( {1 - \eta _{{\mathrm{rad}}}} \right)/{\eta} _{{\mathrm{rad}}}} \right),$$

where IQE is a product of internal radiation efficiency (*η*_rad_) and charge balance efficiency (*η*_inj_)^36^. With ideal (near-unity) *η*_inj_ and *η*_rad_, Eq. 1 becomes:

2 $$ {\mathrm{EQE}}_{{\mathrm{PR,}}\;{\mathrm{max}}{\mathrm{.}}}{\mathrm{ = LEE}}_{\mathrm{0}}{\mathrm{/}}\left( {{\mathrm{LEE}}_{\mathrm{0}}{\mathrm{ + A}}_{{\mathrm{para}}}} \right){\mathrm{,}}$$

Therefore, parasitic absorption is an important limiting factor of LED efficiency, which prevents PeLEDs from achieving the theoretical maximum EQE of 100% for a perfect IQE.

### Optical analysis of PeLED efficiencies

To maximise the benefit of PR, detailed design principles for LED devices can be established with the assistance of optical device modelling^37–39^. In PeLEDs, the presence of re-absorption is a major obstacle to calculations of dipole emission, leading to a divergence of dipole density within the absorbing medium^40^. Although previous studies ignored the re-absorption of the emissive layers to avoid this problem^2,3,14,41^, we resolved the divergence by assuming that (i) internal dipoles are uniformly distributed over the perovskite divided into 20 slices, (ii) only the mesh slice containing the dipole is non-absorbing (i.e., *k* = 0) and the other 19 mesh slices retain their absorption property and (iii) the non-radiative coupling^37,38,42^ of the dipole with other absorbing meshes in perovskite, which is the main reason for divergence, does not affect the external radiation efficiency as the energy remains in the perovskite. The third statement can be understood as virtual PR in the same manner as Förster resonance energy transfer. The transfer matrix formalism (TMF)^37–39,43^ was adopted to calculate the efficiency of each dipole. Full details for our approach and its physical background are provided in Supplementary Figs. 6–9 and Supplementary Notes 4–5.

**Fig. 3:**
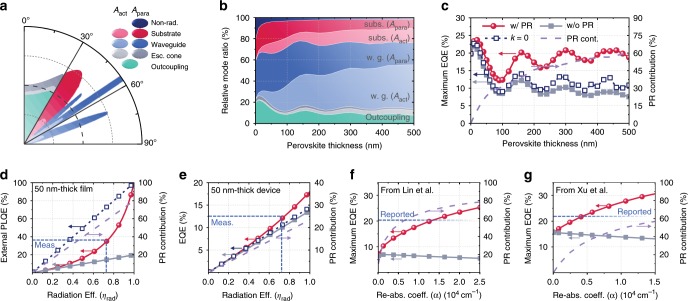
PeLED analysis using TMF modelling. **a** Angular mode analysis for PeLED with a structure of glass/ITO (150 nm)/PVK (10 nm)/PEA_2_Cs_n−1_Pb_n_Br_3n+1_ perovskite (50 nm)/TPBi (50 nm)/LiF (1 nm)/Al. Proportions of active absorption (*A*_act_) and parasitic absorption (*A*_para_) for each mode are represented by unique colours in **a**–**b**. **b** Relative ratios of each mode in PeLED for various perovskite thickness. **c**–**g** Calculated EQEs (of PL or EL) with PR (closed red circles), without PR (closed grey squares) and without re-absorption (open navy squares), as well as relative PR contribution (violet dashed lines) for **c** an ideal PeLED (IQE = 100%) with various perovskite thickness; **d** a 50 nm-thick perovskite film on glass; **e** a PeLED with 50 nm-thick perovskite (*η*_inj_ = 100%); and **f**–**g**, the PeLED structures reported by Lin et al.^4^ and Xu et al.^5^, respectively, with 100% IQE. For **d**–**g** the blue dashed lines compare the graphs with the experimentally measured or reported values. The fact that the calculated *η*_rad_ (75%) of the PeLED **e** is almost the same as that of the neat film **d** indicates that the assumption of *η*_inj_ = 100% is a good approximation, at least for the voltage of 2.6 V where the maximum EQE occurs. See Supplementary Figs. 10–12 for further details.

Our modelling methodology allows precise optical analysis of PeLEDs and quantitative investigation of PR. As the emissive layer ((PEA)_2_Cs_n−1_Pb_n_Br_3n+1_ perovskite) has a refractive index with real part (*n*) of ~ 2 (Supplementary Fig. 6), larger than that of air (1), the optical energy is transferred through either the escape cone, the substrate mode, the waveguide mode, or non-radiative parasitic coupling (also called the plasmon mode, having a complex emission angle) depending on the emission angle^14,38,39^, as shown in Fig. 3a. As trapped modes include photon propagation within the absorbing layers (e.g., ITO, perovskite and Al), non-outcoupled optical energy is eventually re-absorbed if a device has sufficiently large lateral dimensions as observed in spatial EL measurements and Supplementary Fig. 5.

In Fig. 3b, we observe that for thicker emissive layers, the fraction of re-absorption in perovskite (*A*_act_) tends to increase in all radiative modes and the fraction of direct outcoupling decreases. The corresponding maximum EQEs with perfect *η*_inj_ and *η*_rad_ are plotted in Fig. 3c. Although thin perovskite films benefit from the optimised microcavity effect with a local maximum near 20 nm, for thicker perovskites, the maximum EQE without re-absorption (open navy squares) converges to the ray-optical limit of 1/2*n*^2^, which is 13% for *n* equal to 2. The EQE further decreases when re-absorption is considered as a loss mechanism (closed grey squares). On the other hand, if re-absorbed photons recursively generate dipoles with perfect recycling efficiency (*η*_rad_ equal to 100%) (Supplementary Fig. 8), PR allows high EQEs to be obtained. Values ~ 20% are predicted near the local maxima (closed red circles) with PR contributing up to 60% of that value (violet dashed line). Although the experimentally optimised thickness (50 nm) of our devices is closer to the first local maximum at 20 nm, it is noteworthy that a few of the recently reported state-of-the-art PeLEDs^2,4^ use thicker perovskite layers closer to the second local maximum at 160 nm with more PR contribution.

For luminescent films, the measured external PLQEs are typically assumed to be the same as *η*_rad_. However, when re-absorption exists this assumption is no longer valid and the relationship between PLQE and *η*_rad_ is nonlinear, as shown in Fig. 3d. For example, our measured PLQE of 37% (Fig. 1c) corresponds to an *η*_rad_ of 75%, approximately twice the prediction from a linear model. Such a nonlinear relationship also appears for the device, as shown in Fig. 3e. When *η*_rad_ is low, (such as in typical perovskite solar cells under 1 sun illumination) the benefit of PR does not exceed the re-absorption loss and the EQE with PR is similar to that for a non-absorbing perovskite. However, as *η*_rad_ increases, a maximum EQE of 18.0% is reached with the aid of increased PR, which is substantially higher than the value without re-absorption (14.5%), demonstrating the counter-intuitive principle that a reduced Stokes shift enhances outcoupling efficiency in highly radiative devices. This relationship can be used to correctly quantify the IQE and PR contribution in practical devices. For example, for our current device structure giving an EQE of 12.5%, *η*_rad_ is found to be 75% and the fractional contribution from PR is 17.5% (Fig. 3e). This leaves significant further room for enhancement in EQE with increased *η*_rad_ and enhanced PR.

Our model incorporating PR is important to explain the recent successes of achieving high EQE (> 20%) PeLEDs^2–6^, particularly for those based on perovskites with high refractive indices and large re-absorption^3–6^. Fig. 3f–g shows that even with 100% IQE the maximum EQEs from the classical model cannot exceed 9% and 16% for the LED structures reported by Lin et al.^4^ and Xu et al.^5^, who reported EQEs above 20% with 200 nm and 60 nm-thick perovskites, respectively. However, by taking PR into account, maximum EQEs between 20% and 30% are predicted to be achievable, making the measured EQEs reasonable with very large PR contributions above 70% and 30% (i.e., additional EQEs of 14% and 6%) for effective re-absorption coefficients (*α*) above 1.3 × 10^4^ cm^−1^ and 4 × 10^3^ cm^−1^, respectively. Here, we assumed that these *α* values are sufficiently high for re-absorption (and hence PR) to dominate over scattering, at least in the planar devices. Although scattering can in general provide an effective means to extract photons from trapped modes by randomising the propagation angle, we expect it can dominate over PR in some devices having (i) exceptionally rough interfaces or nanostructures;^3,16,25,41^ (ii) large Stokes shifts (e.g., emitters mixed with host materials); or (iii) low radiation efficiency (e.g., solar cells). The study of devices where both PR and scattering are important will be an important goal for future research.

### Advanced schemes to maximise the benefits of PR

As shown in the previous section, the maximum achievable EQE is limited to between 20% and 25% in current PeLED architectures owing to losses from parasitic absorption. Although recently reported devices already approach this upper bound, Supplementary Fig. 10 and Supplementary Note 7 show that the EQE can be further enhanced to between 30% and 50% through materials engineering, which targets perovskites with reduced *n* and increased re-absorption, and transparent conductive electrodes with reduced parasitic absorption.

**Fig. 4:**
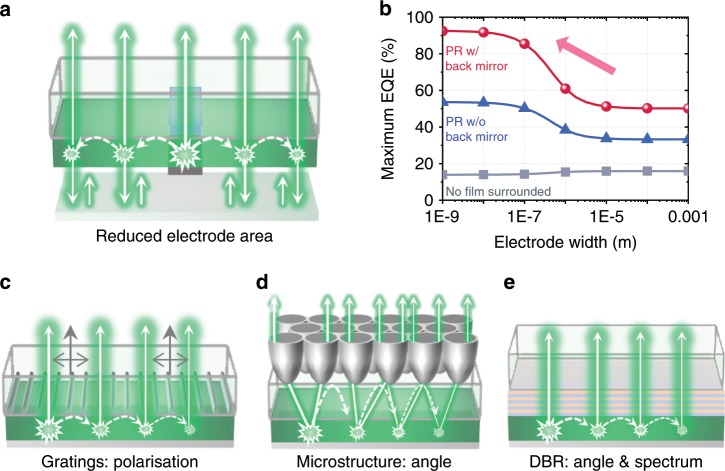
Photonic structure control of PR. **a** A proposed LED architecture consisting of small area electrodes and a large area film, having a reflecting mirror at the bottom. **b** Calculated maximum EQE (IQE = 100%) as a function of electrode width in the proposed systems having PR with back mirror, PR without back mirror (only front re-emission is collected) and no film surrounded (PR only occurs inside the pixel). The device structure in the electrode-covered area was assumed to be same as the one for Fig. 3 (with a 50 nm-thick PEA_2_Cs_n-1_Pb_n_Br_3n+1_ perovskite). **c**–**e** Examples of light property modification using **c** 1D metal nanogrid for polarisation **d** microstructure for angle confinement and **e** DBR for spectrum control.

Ultimately, to achieve ultrahigh EQEs approaching 100%, an improved device architecture design is essential to remove *A*_para_ and maximise the PR effect. Figure 4a shows an example of such a configuration where the emissive area is reduced to a small fraction of the substrate, surrounded by regions without electrodes and associated parasitic absorption. Once a photon escapes the small region with injection electrodes, parasitic absorption is avoided, and in the limit of high *η*_rad_ all photons will eventually be outcoupled. A back mirror (not necessarily conductive) with high reflectivity ensures all photons are extracted in the forward direction. Figure 4b shows that a maximum EQE of over 90% is achievable in this system with an electrode width of < 30 nm. This implies that dedicated design of nanostructured electrodes will bring further breakthroughs in LED efficiency with the aid of PR, in addition to their scattering and plasmon effects^3,16,44,45^. Moreover, although this calculation assumed dipole generation immediately beneath the electrode, charge carrier diffusion can improve the effective injection area of LEDs with nanostructured electrodes, without increase of parasitic absorption loss. Accordingly, the benefit of efficient PR can be achieved without damaging the areal density of light emission, by setting the distances between nanostructured electrodes to be similar to the diffusion length of charge carriers.

Not only the quantity of photons, but also properties of emitted photons can be optimised utilising PR. When emitted photons are filtered by a selective transmitter, photons having the non-desired property can be recursively recycled with the randomised property within the emitting film. In the limit of perfect PR and zero parasitic absorption loss, a 100% filter transmission could be achieved for the desired property. Figure 4c–e illustrate more examples of light property optimisation—a 1D metal nanogrid^46,47^ that aligns polarisation; an angle confinement microstructure^48,49^, which confines emission angle; and a distributed Bragg reflector^35,50,51^, which narrows both emission angle and spectrum. Polarised emission will be especially useful in display applications, reducing the 50% efficiency losses typically encountered in the circular polariser needed to achieve high contrast ratios.

## Discussion

The study illuminates the current state and potential of PR in PeLEDs, demonstrating how the efficiency and properties of light emission are enhanced. Spatially resolved luminescence measurements have shown lateral propagation of emission over 300 μm in perovskite films, demonstrating efficient PR in the material. By recycling the photons trapped in the substrate and waveguide modes, PR has been shown to significantly contribute to the light outcoupling in luminescent perovskite optoelectronic devices having high *η*_rad_, such as PeLEDs. Schemes incorporating reduced electrode area and various filtering structures have been proposed to maximise the benefit of PR in current PeLED architectures.

## Methods

### Preparation of perovskite precursor and film

The perovskite precursor was prepared by dissolving 110.1 mg PbBr_2_ (Sigma-Aldrich), 63.8 mg CsBr (Sigma-Aldrich) and 67.46 mg PEABr (Dyesol) in 1 mL of anhydrous dimethyl sulfoxide (Sigma-Aldrich). The precursor solution was stirred using a magnetic stir-bar for ~ 12 h at room temperature before use. For the experiment of spatially resolved PL, the perovskite film was prepared by dropping 20 μL perovskite precursor onto a PET substrate spinning with 2000 rounds per minute (RPM).

### Fabrication of device

To measure spatial EL, a pre-patterned ITO substrate (15 Ω sq^−1^, Colorado Concept Coatings) was cleaned using ultrasonication in acetone and isopropanol for 15 min, respectively, and then dried with a nitrogen blow gun, after which the substrate was treated under oxygen plasma etching (forward power, 300 W; reflected power, 0 W, 10 min). The ITO substrate was then transferred to a nitrogen-filled glovebox. PVK was spun from chlorobenzene solution (6 mg mL^−1^) at RPM 3000 and was annealed at 120 ˚C for 10 min. Subsequently, the perovskite was deposited from the precursor to form a ~ 50 nm layer using 4000 RPM, and was annealed at 70 ˚C for 8 min. Finally, 50 nm TPBi, 1 nm of LiF and 80 nm of Al were sequentially evaporated through a shadow mask at a pressure of 10^−6^ mBar. The device was encapsulated with UV epoxy (NOA81, Thorlabs)/cover glass to avoid exposure to oxygen and moisture and degradation during measurement.

### EQE characterisation

A Keithley 2400 source-metre was used to obtain the current density–voltage (*J*–*V*) characteristics. The relative angular characteristics of the emission were measured by rotating the device at 20 cm from the silicon photodiode. The luminances and EQEs were calculated by applying the relative angular characteristics to the front emission measured using a silicon photodiode of which spectral response is known. The LED measurement setup was previously cross-checked against a third-party industrial laboratory^8^.

### PL and PLQE measurement

PL was measured with an integrating sphere according to the method previously reported^52^. A continuous wave 405 nm diode laser with a beam size of 0.3 mm^2^ was used for excitation. Photons were collected using an Andor iDus DU420A Si detector with an integration time of 0.02 s (for excitation > 10^18^ m^−2^ s^−1^) or 0.2 s (for excitation < 10^18^ m^−2^ s^−1^).

### UV–vis and PLE measurement

Absorption spectra of thin-films deposited on fused silica substrates were measured using Hewlett-Packard 8453 UV−vis spectrometer with blank substrate correction. The photoluminescence excitation (PLE) spectrum was obtained by measuring a perovskite-coated glass with FLS980-S2S2-sm (Edinburgh Instruments Ltd.) equipment. Each measured value was normalised to incident excitation intensity. The excitation wavelength was scanned while detecting the fixed wavelength of 525 nm (for a single spectrum, dwell time of 0.20 s per each point) or various wavelengths (for mapping, dwell time of 0.10 s per each point) with a detection range of 1 nm.

### Spatially resolved PL/EL

The spatially resolved PL was measured using a WITec Alpha RAS system. The sample was rested on a XYZ piezo stage of the microscope and a black aperture was inserted below the substrate to prevent any unexpected light pathway such as diffusion at the excitation lens and reflection at the bottom stage. The film coated on the top of the PET substrate was excited by a 405-nm c.w. laser (Coherent CUBE), focused using a × 40 objective lens (NA = 0.60) from the bottom side and the PL was collected in transmission geometry using a × 20 objective lens (NA = 0.40) above the top side. The lateral distance between the excitation and collection was controlled by WITec ScanCtr Spectroscopy Plus software. The signal at each point was obtained by averaging several repetitive measurements with the integration time of 1 s. The signals for 505 nm and 525 nm were collected by integrating the ranges of 500–510 nm and 520–530 nm, respectively. The spatially resolved EL has been measured using the same setup by replacing the excitation light with an applied voltage of 4 V. The full device with a structure of glass/ITO (150 nm)/PVK (10 nm)/perovskite (50 nm)/TPBi (50 nm)/LiF (1 nm)/Al was measured. As photon flux is diluted at the far distance, the far range signal was constructed by averaging 36 measurements with integration time of 26 s to secure sufficient signal-to-noise ratio. All the measured quantities of the signal were divided by the integration time. Refer to the SI for further details of reliability issues related to this measurement.

### Optical modelling

The TMF^37–39,43^ was used to calculate the emissive characteristics in PeLEDs having a structure of glass (*n* = 1.5, thick)/ITO (150 nm)/PVK (*n* = 1.71, 10 nm)/perovskite/TPBi (*n* = 1.76, 50 nm)/Al, where *n* indicates the refractive index of each layer. Refractive indices of ITO^53^ and Al^54^ were obtained from the literature and that of perovskite was obtained by fitting the Kramers–Kronig relation^55^ for the measured transmission and PLE spectrum (refer to Supplementary Fig. 6). As the imaginary part (*k*) of the refractive index in perovskite sharply varies within the range of the luminescence spectrum, we performed calculations throughout the wavelength range from 480 nm to 560 nm and integrated (Supplementary Fig. 9), with the assumption that internal dipole radiation has a spectrum identical to the measured PL under 405 nm excitation (Fig. 1a). The active layer was divided into 20 mesh slices and dipoles were assumed to be uniformly distributed to the mesh slices as described in Supplementary Fig. 7a. Based on the calculated internal electromagnetic fields from TMF, we calculated Poynting vectors (i.e., energy fluxes) at each interface having directions normal to the interfaces^39^. Then, for a given dipole with a given emission angle, the re-absorption in each layer can be obtained from the differences between the Poynting vectors at the front and back interfaces. We calculated quantities of light emission and re-absorption by sweeping dipole properties of emission angle, wavelength, orientation and polarisation. The results of mode analysis have been obtained by integrating those swept quantities.

### Modelling for the reported devices

Reported structures of glass (*n* = 1.5, thick)/ITO (*n* = 1.80 + 0.020*i*, 150 nm)/PEDOT:PSS (*n* = 1.54, 40 nm)/perovskite (*n* = 2.2^13,15,56^ and *k* varies, 200 nm)/poly(methyl methacrylate) (PMMA, *n* = 1.49, 8 nm)/C_37_H_26_N_6_ (B3PYMPM, *n* = 1.85, 40 nm)/Al (*n* = 0.86 + 6.41*i*, thick) and glass (*n* = 1.5, thick)/ITO (*n* = 1.51 + 0.027*i*, 150 nm)/ZnO:PEIE (*n* = 1.58, 40 nm)/perovskite (*n* = 2.5^15^ and *k* varies, 60 nm)/poly(9,9-dioctyl-fluorene-co-*N*-(4-butylphenyl)diphenyl-amine) (TFB, *n* = 1.57, 30 nm) /molybdenum oxide (MoO_3_, *n* = 1.98, 5 nm)/Au (*n* = 0.18 + 5.11*i*, thick) were used for the devices of Lin et al.^4^ and Xu et al.^5^, respectively. For simplicity, a monochromatic model was used for the wavelength (λ) of 525 nm and 800 nm, respectively, varying effective *k* values (= αλ/4π) of perovskite. Refractive indices at each wavelength were obtained from various literatures^53,54^.

### Modelling for the proposed concept with reduced injection electrode area

In the proposed system with reduced injection electrode area (Fig. 4a–b), we assumed that a full PeLED (the same as that used in Fig. 3) with an infinite length and finite width is positioned at the centre of a 1.1-mm-thick system with infinite lateral dimensions. The radiation efficiency of perovskite and the reflectivity of the back mirror are assumed to be 100% and 98%, respectively. In the electrode-covered area, the emitted dipole was assumed to have a mode fraction shown in Fig. 3b, ignoring possible optical effects from nanopatterned electrodes. In the pure-film area, it was assumed to have 50% forward emission and 50% backward emission. Then, if a photon escapes the electrode area, it can be recycled with efficiencies of 99% (50% × 100% + 50% × 98%, for PR with back mirror), 50% (for PR without back mirror) and 0% (for no surrounding film), respectively. The 1D simulation domain for the electrode-covered area was sliced into 1 nm size meshes and dipoles were assumed to be uniformly distributed over this area. When a photon in the escape cone is re-absorbed by perovskite, it was assumed to be re-emitted at the same mesh. When light is trapped by the waveguide mode, it decays exponentially in both left and right directions, with a decay constant of *α*_act_ + *α*_para_, where *α*_act_ indicates a perovskite re-absorption constant (*α*_act_ = 1.09 μm^−1^ from the average of the measured absorption coefficient of our perovskite) and *α*_para_ indicates a constant for parasitic absorption loss, assumed to be *α*_para_ = *α*_act_ × *A*_para_/*A*_act_ in the waveguide mode. All photons in the substrate mode can directly escape the electrode because their single-trip propagation distance (> 2 mm) is longer than the electrode widths used in the calculation. The calculation was recursively performed until no photon remains in perovskite of the electrode-covered area.

## Supplementary information

Supplementary Information

## Data Availability

The data underlying each Figure in this paper are available at (10.17863/CAM.46319).
