# Supplementary material for: The role of photon recycling in perovskite light-emitting diodes
Source: Nat Commun. 2020 Jan 30;11:611. doi: 10.1038/s41467-020-14401-1 (PMC6992794; doi:10.1038/s41467-020-14401-1)
Supplement: Supplementary file 1 — Supplementary Information [file 41467_2020_14401_MOESM1_ESM.pdf]

Supplementary Information

The role of photon recycling in perovskite light-emitting diodes

Changsoon Cho, Baodan Zhao, Gregory D. Tainter, Jung-Yong Lee, Richard H. Friend, Dawei Di, Felix Deschler, Neil C. Greenham

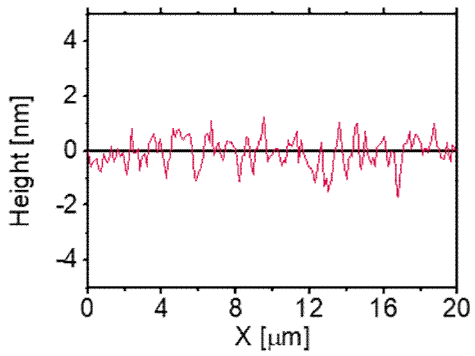

Supplementary Figure 1. Relative height of a perovskite film studied in the manuscript, scanned with atomic force microscopy (AFM). The root mean square (RMS) roughness of our perovskite film was calculated to be 0.55 nm. Due the film's smoothness, light scattering is minimized and we therefore attribute the majority of the measured scattering signal in the spatial PL to the haze of our PET substrate.

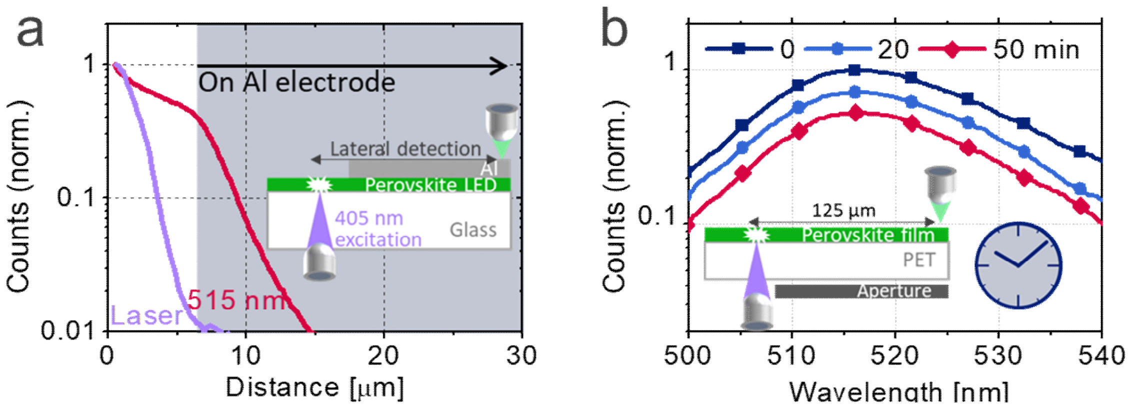

Supplementary Figure 2. Reliability test for the spatially resolved PL. a, Spatially resolved laser and PL signals for a perovskite LED covered with a patterned Al electrode (shaded region) to quantify the spatial resolution of the detecting lens. Curves detailing the decay of laser (purple curve) and PL intensity (515 nm, red curve) are presented. We observe that the PL signal is observable even over the Al electrode, though the change in the rate of decay in collected PL intensity demonstrates that this contribution decays more rapidly with distance than contributions from PR. **b,** Measured degradation of the PL spectrum at a distance of 125 μm on a perovskite film as a function of exposure time. See Supplementary Note 1 for further details.

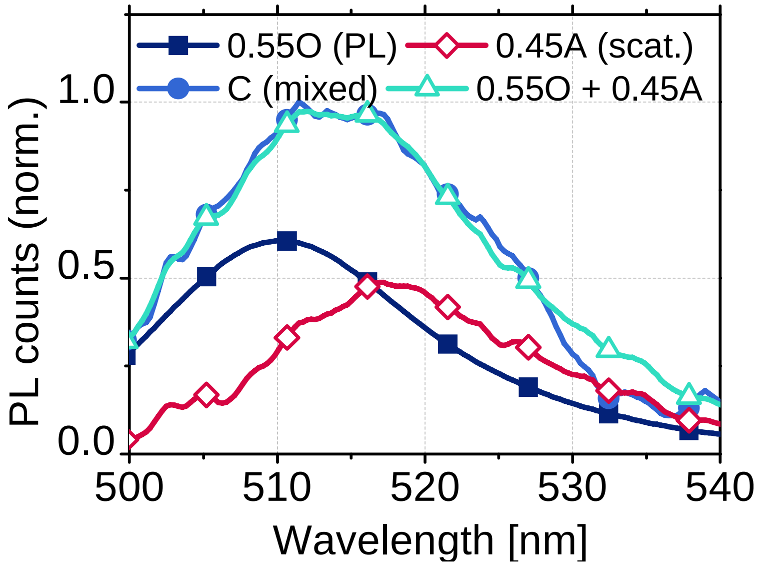

Supplementary Figure 3. Spectral analysis of the signal measured at long distance from the excitation. Spectra O, A, and C were measured at the original excitation point (O, at 0 μm in Figure 2c, PL-only) and long distances without (A, at 265 μm in Fig. 2c, scattering-only) and with (C) perovskite covering, respectively, as shown in Figure 2b-c. The broad spectrum C (at 310 μm) can be fitted by combining 55% PL signal (O) and 45% scattering signal (A).

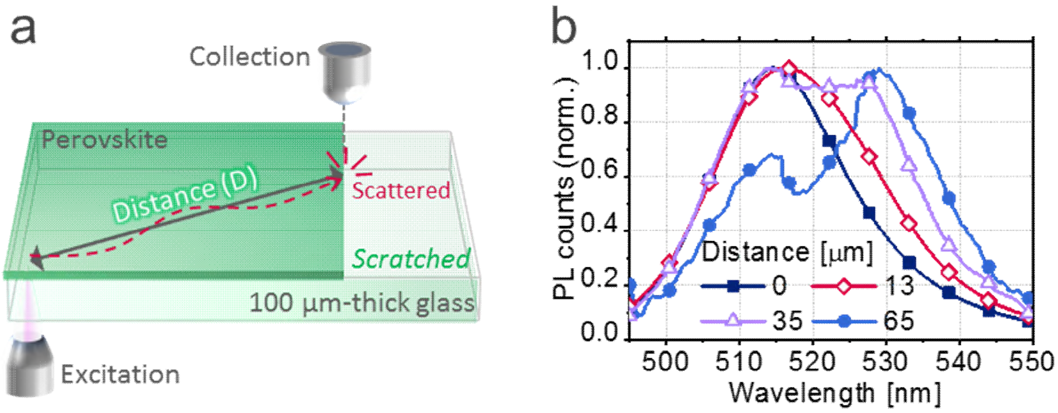

Supplementary Figure 4. Laterally measured spectra of photons trapped in the waveguide mode. a, Schematic representation of spatially-resolved PL, collecting photons at the edge of a perovskite film. **b,** Measured PL spectra at the edges (at 13, 35, and 65 μm), varying the distance from the excitation at 0 μm. See Supplementary Note 2 for further details.

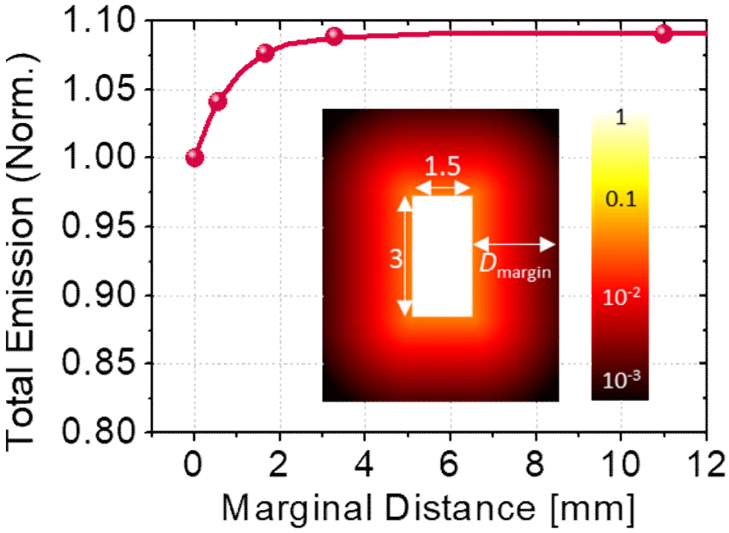

Supplementary Figure 5. Diffused emission in the LED we measured. Estimated total emission with various marginal distances (D_{margin}s) from a 1.5 mm × 3 mm size emitting pixel (positioned at the centre) to the edge of the substrate in the PeLED measured in Fig. 2d ($T_{substrate}$ = 1.1 mm). The value was normalised to the total emission of a system without marginal distance, which is typically assumed in the measurement. (inset: spatial emission profile for a domain with D_{margin} = 2.2 mm). See Supplementary Note 3 for further details.

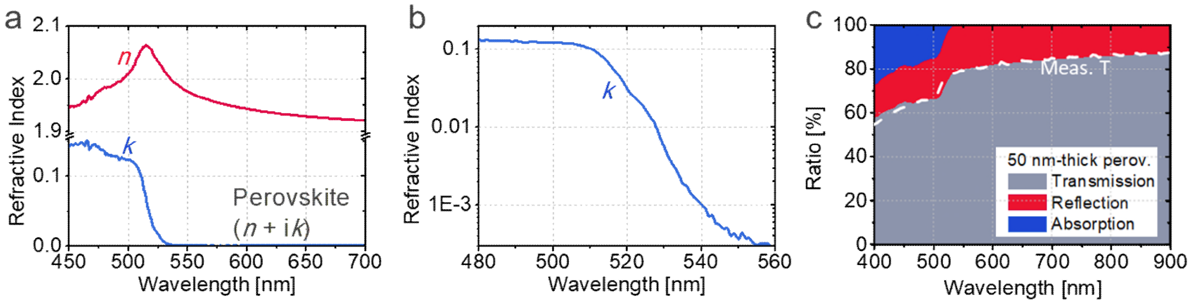

Supplementary Figure 6. Optical constants of perovskite. a, Real (*n*) and imaginary (*k*) parts of refractive index of perovskite used in the manuscript. **b,** *k* in log scale. **c,** Calculated transmission, reflection, and absorption ratios of 50 nm-thick perovskite, compared to the measured transmission spectrum (with an air reference). See Supplementary Note 4 for further details.

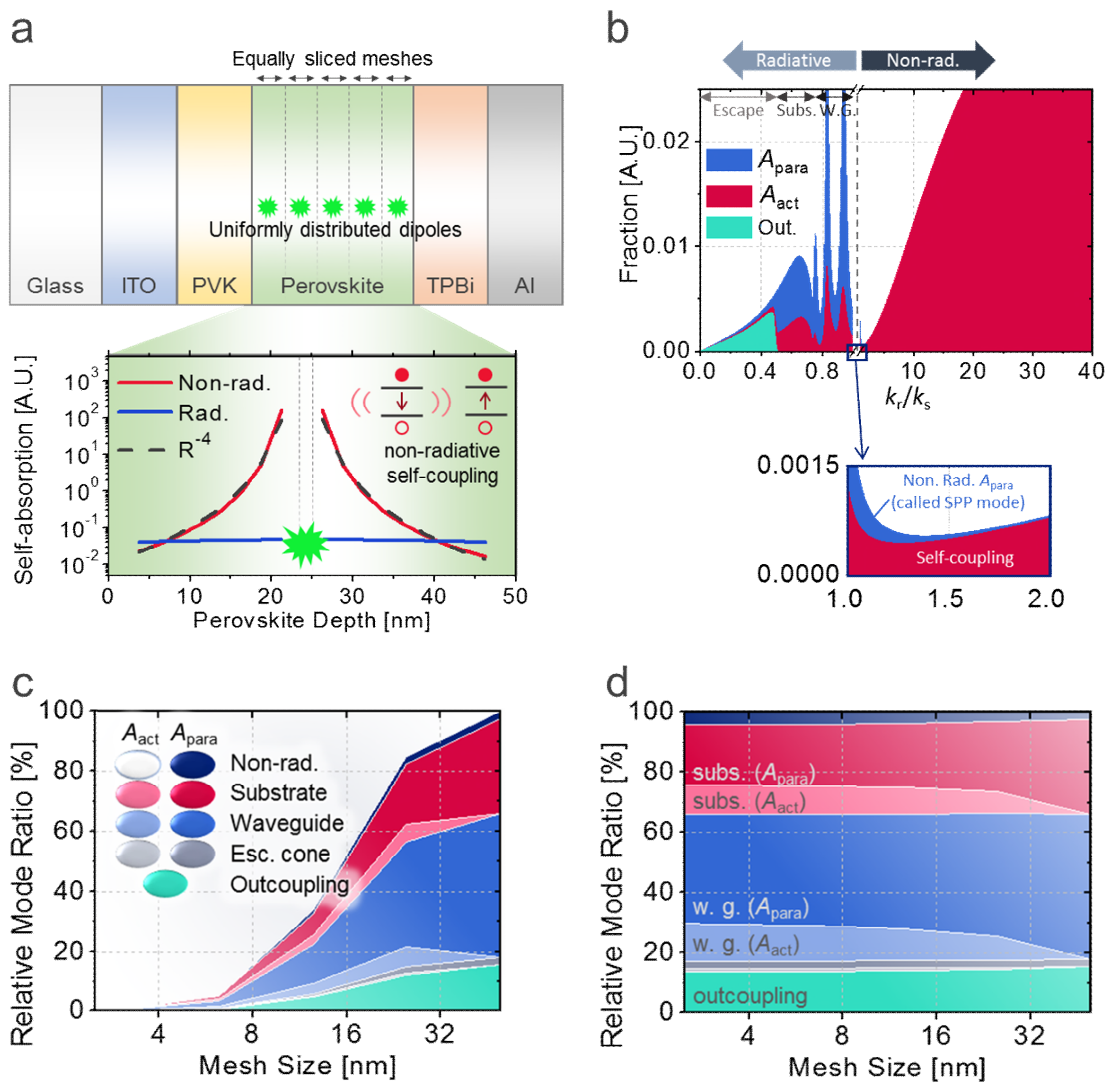

Supplementary Figure 7. Detailed modelling results for a 50 nm-thick perovskite LED. a, Simulated LED structure with a uniformly sliced perovskite emissive layer, having uniformly distributed dipoles. The graph represents the re-absorbed energy profile in perovskite through non-radiative and radiative coupling when a dipole is positioned at the middle of the layer. The non-radiatively coupled energy is shown to be proportional to R^{-4}, where R indicates the distance to the dipole. **b**, Relative power emission or dissipation as a function of the normalised in-plane vector (k_r/k_s), obtained by integrating the whole spectrum and positions of dipoles. (mesh size: 2.5 nm) **c-d,** Relative mode ratios **c,** including and **d,** excluding non-radiative self-coupling (represented by white region) as a function of mesh size. See Supplementary Note 5 for further details.

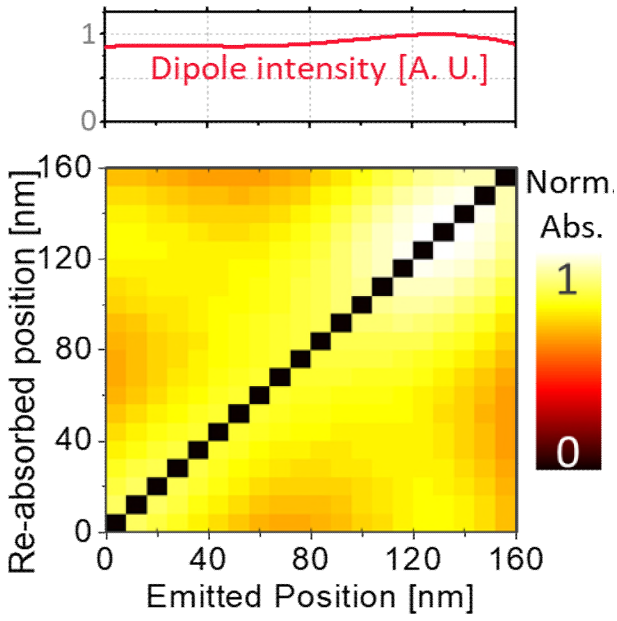

Supplementary Figure 8. Normalised spatial profile of re-absorption for dipoles emitting at various positions in 160 nm-thick emissive layer of a PeLED. The top graph represents the calculated total dipole intensity as a function of emitted position (x-axis is shared with the bottom image). For our modelling, as described in the manuscript, the perovskite layer is sliced into 20 meshes with equal size. Dipoles are assumed to be uniformly distributed over the depth, but the properties (spectrum, intensity, orientation, and polarisation) of each are not constant, because they are coherently affected by the optical environment (e.g. microcavity effects), as shown in the top graph, integrating all the emitted power at each emitted position. The spatial profile in the main panel shows that the strongest absorption tends to appear at the nearest meshes to the radiation and decreases as the distance increases. For the PR calculation in the manuscript, it was assumed that a new dipole is generated with an efficiency of η_{rad} where a dipole is re-absorbed. So the internal profile of optical energy varies along the recursive calculation and it continues until no energy remains in the emissive layer. Such varying dipole profile makes the resulting maximum EQE (Figure 3c) slightly different from Equation 2 neglecting the spatial dependence of LEE_0, A_{act}, and A_{para}.

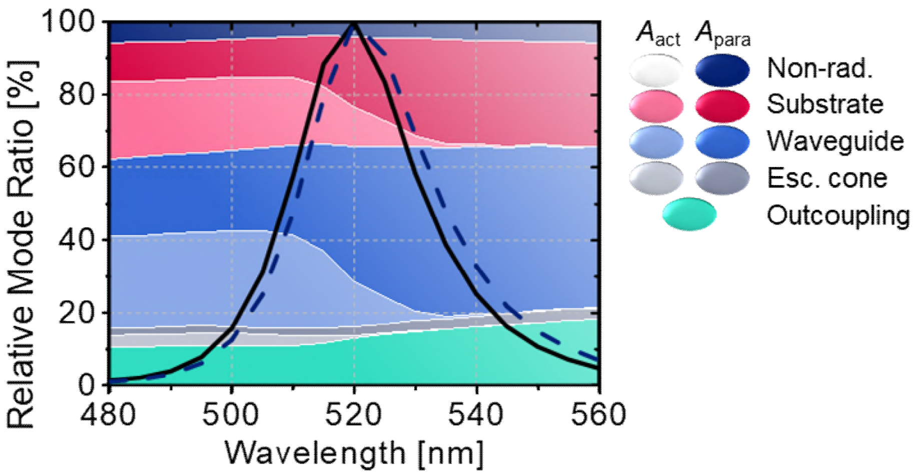

Supplementary Figure 9. Dipole mode analysis at each wavelength. Solid and dashed lines indicate the spectra of internal dipole (from Figure 1a) and outcoupled light (calculated from the internal spectrum), respectively. For the TMF calculation, a wavelength range from 480 nm to 560 nm was integrated assuming that the dipole has an emission spectrum the same as the measured photoluminescence. The figure shows the calculated mode ratios as a function of wavelength in a PeLED having 50 nm-thick perovskite. Because of the re-absorption loss, the direct outcoupling ratio is shown to be higher in the red region, while photons in blue region have further chances to be recycled through the relatively large re-absorption (A_{act}) of perovskite. Accordingly, the spectrum of outcoupled light is shown to be slightly red-sifted relative to the internal emission.

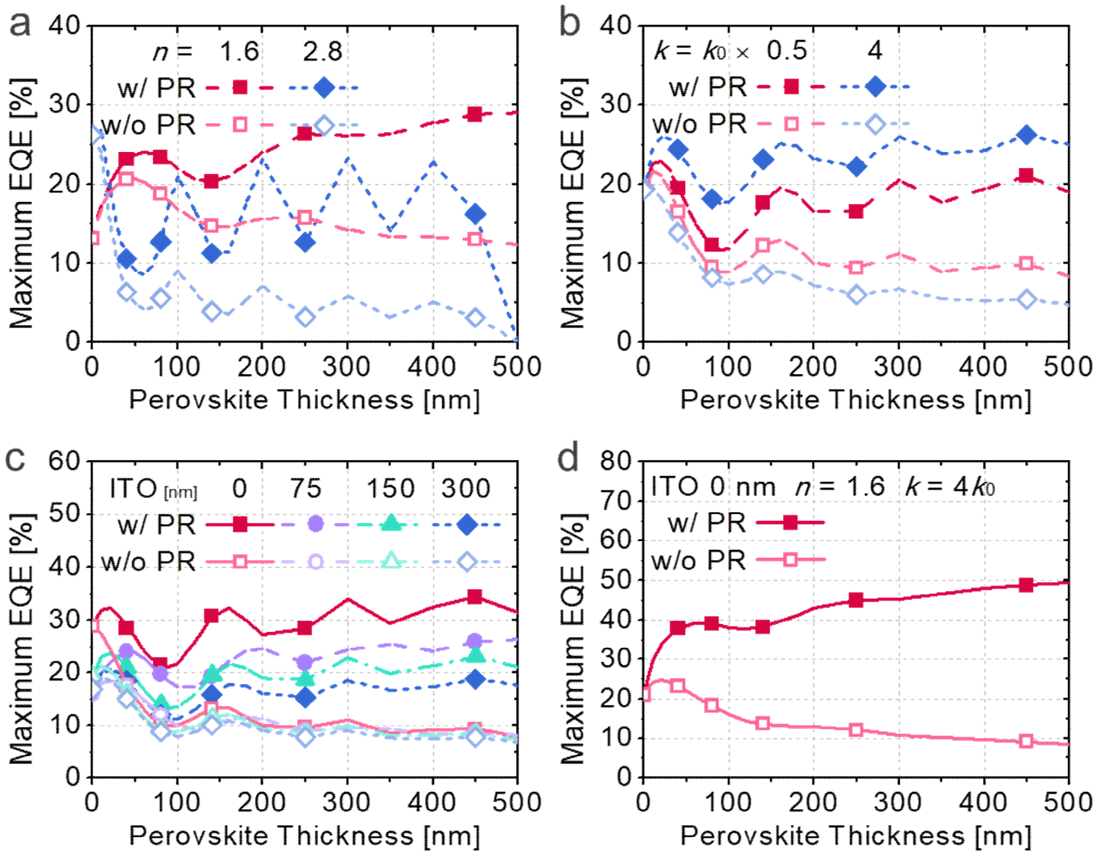

Supplementary Figure 10. Optimisation of PeLEDs with PR. Maximum EQEs with and without PR for PeLEDs with various thickness used in the manuscript, **a-b,** changing refractive index of perovskite to **a,** 1.6 + i$k_0(\lambda)$ or 2.8 + i$k_0(\lambda)$ and **b,** $n_0(\lambda)$ + i(0.5$k_0(\lambda)$) or $n_0(\lambda)$ + i(4$k_0(\lambda)$), and **c,** changing ITO thickness to 0 ~ 300 nm. **d,** Those for the best device having refractive index of 1.6 + i(4$k_0(\lambda)$) without ITO. See Supplementary Note 6 for further details.

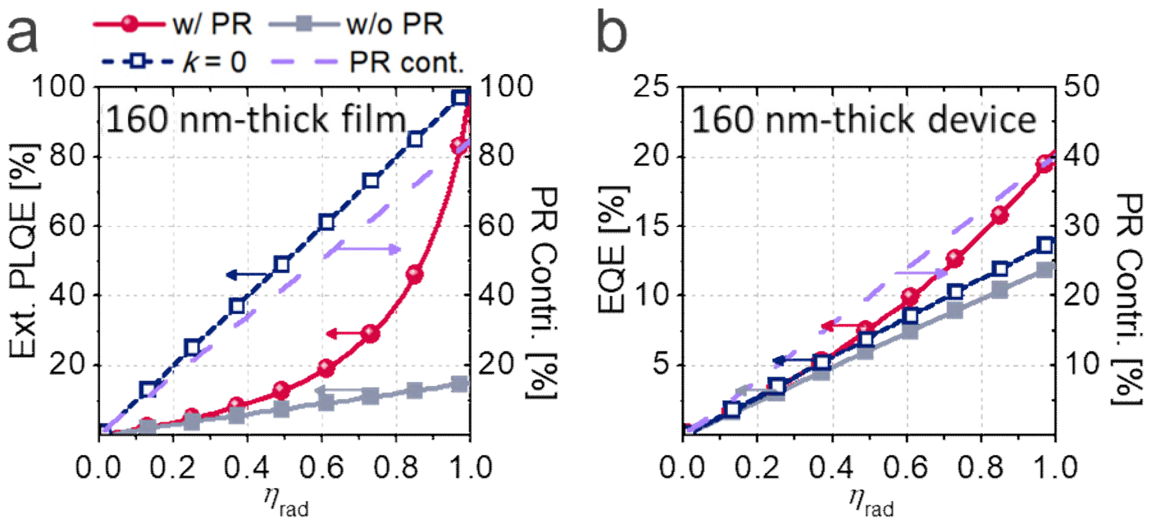

Supplementary Figure 11. Calculated EQEs (of PL or EL) with PR (filled red circles), without PR (filled grey squares), and without re-absorption (open navy squares), as well as relative PR contribution (violet dashed lines) for **a,** a 160 nm-thick perovskite film on glass and **b,** a PeLED with 160 nm-thick perovskite (η_{inj} = 100%). Fig. 3d-e show the calculated EQEs as a function of η_{rad}, for the film and device with 50 nm-thick perovskite, according to our experimental condition. While the practical PR contribution is relatively small in such thin films, many of the present state-of-the-art devices [4,8] use thicker films around 200 nm thickness having larger PR effects. The figure shows the results of the same calculation for 160 nm-thick perovskite, corresponding to the second peak of EQE in Figure 3c. Compared to Figure 3d, the graph of external PLQE becomes more curved, resulting in a larger difference between the external and internal radiation efficiencies. In (b), the maximum PR contribution is shown to increase to 40.4%, which is 72.6% times larger than 23.4% in Figure 3e.

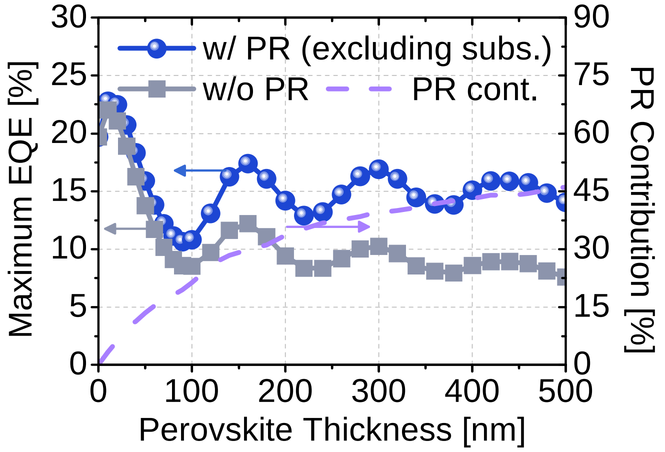

Supplementary Figure 12. PR effects excluding substrate mode. Calculated maximum EQEs with PR (blue circles, excluding PR for the substrate mode) and without PR (grey squares), as well as relative PR contribution (violet dashed line) for an ideal PeLED having IQE = 100% with various perovskite thickness. In the manuscript, we assumed that the device is large enough to reabsorb all the photons in the substrate mode during the lateral propagation according to the calculation in Supplementary Figure 5. However, in display applications, such long-range diffused emission is not desirable due to the reduced sharpness and only the photons in the escape cone and waveguide mode have a chance to be recycled within small size pixels having widths of < 10 μm. The Figure shows the calculated PR effects excluding the substrate mode (i.e. only photons in the escape cone and waveguide mode can be recycled). The maximum EQEs for the thicknesses of 20 nm and 160 nm are shown to be 22.5% and 17.4%, respectively, which are between the values with complete PR (red line of Figure 3c) and without PR (grey line). Since A_{act} in the substrate mode is small for thin perovskite in Figure 3b, reduction of the maximum EQE is relatively smaller for 20 nm thickness when the substrate mode is excluded.

Supplementary Note 1

Reliability issues of the spatially resolved photoluminescence (PL) measurement

Since the spatially resolved PL and EL detect weak signals at long distances from the excitation, the analysis must be exceptionally careful to avoid possible misinterpretation. While we attributed the origin of broad spatial PL and EL shown in Figure 2 to recycling and scattering of trapped photons, we performed additional control measurements to exclude other possible pathways that might cause such a broad signal.

First, as shown in Supplementary Figure 2a and its inset, we excited a perovskite region near the edge of a perovskite LED pixel covered with a 100 nm-thick Al electrode, and collected the signal as a function of distance. In the uncovered region, while the detected laser profile rapidly decays with a full-width at half-maximum (FWHM) of 3 μm, that of PL measured for 515 (±5) nm is shown to be broader, possibly due to the nonlinear relationship between PL and excitation intensity shown in Fig. 1c. In the Al-covered region, the PL signal starts to decay rapidly (ten-fold per 4.6 μm), which can be regarded as the lateral resolution of the detection system as no PL underneath the focal point can outcouple through the Al electrode. This spatial resolution supports our attribution of PL signal to PR in Fig. 2b, where we observe that PL at 505 nm decays by only a factor of 3 moving from an excitation—collection separation of 210 μm to 310 μm. The sharp detection resolution is also shown in the rapid decay of 505 nm signal near separations of 230 μm, at the edge of the scratched region, in Fig. 2b.

Although the broad spatial PL appears near the excited spot in Supplementary Figure 2a, we must confirm that photon recycling (PR) and scattering are the primary origins of this effect. To block the pathway for externally-diffused excitation light, we inserted a black aperture as depicted by a grey box in the inset of Supplementary Figure 2b. Nevertheless, as our thin perovskite film does not fully absorb the laser, a small portion of the laser may remain and circulate in the system by Fresnel reflection or scattering. Since PL from the diffused laser has the same spectrum as that from PR, there

is no way to spectrally distinguish the signals from diffused laser and PR. For example, with our excitation lens having an (NA) value of 0.6, the 405 nm photons have a propagation angle up to 24° in the substrate (n = 1.5) and can reach the lateral distance of ~0.87 T if they bounce on the top and bottom surfaces of the substrate once by Fresnel reflection (T = substrate thickness). This is why we chose a thin PET substrate (T = 23 μm) and long distance (>200 μm) for the analysis in Fig. 2b-c to minimise the influence of such laser diffusion. In order to gain further confidence that the PL signal originates from PR rather than diffused laser light, we measured PL at 125 μm over time and observed degradation of in the emissivity of the film after laser exposure for 50 minutes. Here, we assume that density of absorbed photons at 125 μm is low enough to prevent degradation and the PLQE in this region is not changed (i.e. only the material near the direct illumination undergoes degradation) during the measurement as our perovskite film is stable in dark conditions for several days. In the result, Supplementary Figure 2b shows that the signal at 125 μm is clearly reduced over the whole spectrum with increasing exposure time. This indicates that the signal in this region is subject to the PL intensity at the excited region, which undergoes degradation due to strong light exposure, rather than the diffused laser intensity, which is constant throughout the measurement. Moreover, unlike the signal from the diffused laser, of which spectrum should be the same as “O” in Fig. 2c, the signal from trapped photons (i.e. PR + scattering) should have a broader and red-shifted spectrum similar as “C” in Fig. 2c. Hence, if the diffused laser had a meaningful influence on the signal, the signal would be expected to blue-shift with exposure time, as the signal from trapped photons is more sensitive to the degradation of the originally excited region. Therefore, the maintained spectral characteristic without such blue-shift shown in Supplementary Figure 2b verifies that the side effects such as laser diffusion are negligible compared to scattering and recycling of the trapped photons we studied.

Supplementary Note 2

Spectra of photons trapped in the waveguide mode

In Figure 2c, we discussed the highly red-shifted emission from the edge side (signal B at 290 μm) of the perovskite film, which came from the photons trapped in the waveguide mode. For the deeper investigation, we measured PL at edges with shorter distances from the excitation as shown in Supplementary Figure 4a. Supplementary Figure 4b clearly shows that, while the PL shape at the blue region is little changed, additional peaks appear at the red region and tend to be red-shifted along the distance variation of 13, 35, and 65 μm, Unlike Figure 2, this measurement was performed on a glass substrate much thicker (100 μm) than the investigated distances. Then, PR from substrate mode can be mostly ignored and the result can be explained with photons trapped in the waveguide mode (i.e. propagating inside the film), which can be scattered out at the edges. The photons in the waveguide mode undergo intensive re-absorption during propagation, especially for short wavelengths with higher absorption coefficient. This measurement experimentally demonstrates such a red-shift in the waveguide mode, more clearly than the signal B in Figure 2c, which is mixed with other signals related to substrate mode. It should be noted that the quantitative ratio between original PL (near 515 nm) and scattered signal (> 520 nm) in Supplementary Figure 4b does not have a physical meaning as the edge-scattering efficiency is not consistent for each measurement.

Supplementary Note 3

Quantitative estimation of the diffused emission in practical LEDs

Unlike the classical LED model, in PeLED, our spatial EL measurement has demonstrated that photons can be outcoupled even at long distances from the excited pixel, as shown in Figure 2d. While PR of the waveguide mode has only a short propagation distance on a scale of $1/\alpha \sim 1$ μm (α: re-absorption coefficient of perovskite), the measured long-range diffused emission can be attributed to recycling of photons trapped in the substrate, either by total internal reflection (TIR) or Fresnel reflection. Knowing the dimension of such diffused emission would be helpful in the design of device architecture and precise measurement of efficiencies.

Using the measured spatial EL, we roughly estimated the profile of diffused emission. For estimation, we assume that (i) the emission intensity at a given spot is determined by the shortest distance (D_{min}) to the excited pixel and (ii) the contour integration along the same D_{min} is proportional to $\exp(-\alpha_{eff} \times D_{min})$, with a fitted effective decay constant α_{eff}. Then, the spatial emission intensity can be calculated by:

$$I(x,y) = I_0 \exp(-\alpha_{eff} \times D_{min}(x,y))\ (\textit{pixel boundary length}) / (\textit{contour length of the constant } D_{min})$$

$$= I_0 \exp(-\alpha_{eff} \times D_{min}(x,y))\ (2W_{pixel} + 2L_{pixel}) / (2W_{pixel} + 2L_{pixel} + 2\pi\, D_{min}(x,y)) \quad (1)$$

for a rectangular excited pixel with a width of W_{pixel} and length of L_{pixel}. According to Figure 2d, when the emission inside the pixel is assumed to be 1, those for D_{min} = 37 μm and 1.8 mm are 0.0449 and 0.00315, respectively, with a pixel having W_{pixel} = 1.5 mm and L_{pixel} = 3 mm. Then, we could obtain the fitted constants of I_0 = 0.0479 and α_{eff} = 1.06 mm^{-1}. The resulting spatial profile is plotted in the inset of Supplementary Figure 5. Then, we can calculate the total light emission of a rectangular domain, which corresponds to the substrate size in practical measurements, varying the marginal distance (D_{margin}) from the pixel to the domain boundary. It should be noted that the diffusion of emission caused by the substrate mode is proportional to the thickness of substrate ($T_{substrate}$), which is 1.1 mm in our device. As shown in Supplementary Figure 5, the region out of the pixel is shown to

add total emission as D_{margin} increases, and the enhancement saturates at 9.04% for infinite D_{margin}. In practical cases, additional emissions of 6.38%, 8.28%, and 9.02% (relative to the original emission) are expected from the region out of the pixel for D_{margin}s of $1T_{substrate}$, $2T_{substrate}$, and $5T_{substrate}$, respectively. In our device having a 1.2 cm × 1.2 cm size substrate, D_{margin} is approximately within $2T_{substrate}$ ~ $6T_{substrate}$ depending on the direction, and only small portion of trapped photons would escape the device before being re-absorbed.

Supplementary Note 4

Refractive index of perovskite used in the optical simulation

The re-absorption coefficient plays a key role in accurate calculation of LED structures with small Stokes shifts. While typical optical measurements such as ellipsometry and UV-visible spectroscopy may provide some information about absorption coefficients, the PL excitation (PLE) measurement shown in Figure 1a provides optical characterisation with high sensitivity even in the region of re-absorption with a low absorption coefficient. Therefore, we obtained the extinction coefficient (*k*) of the refractive index from the PLE spectrum ($PLE(\lambda)$) by assuming that the measured PLE is proportional to the film absorption, at least in the narrow spectral range of our interest, as shown below:

$$PLE(\lambda) = P_0 \times (1 - \log(-\alpha(\lambda) \times thickness)) \quad (2)$$

$$k(\lambda) = \alpha(\lambda) \times \lambda / 4\pi = - \lambda \times \exp(1- PLE(\lambda)/ P_0) / (4\pi \times thickness) \quad (3)$$

The real part (*n*) of refractive index can be obtained from *k*, by applying a closed-form Kramers-Kronig relation fixing a single value at an arbitrary wavelength ($n(\lambda_0) = n_0$).[1] Then, the whole spectrum of $n(\lambda) + ik(\lambda)$ can be represented by only two fitting parameters of P_0 and n_0. We performed the transfer-matrix formalism (TMF) for a single 50 nm-thick perovskite film coated on a glass substrate optimising P_0 and n_0 to match the calculated and measured transmissions. The resulting *n*, *k* values and corresponding transmission are shown in Supplementary Figure 6. We obtained the precise *k* value in the re-absorption region with a sensitivity below 10^{-3} as shown in Supplementary Figure 6b.

Supplementary Note 5

Virtual photon recycling: non-radiative self-coupling of dipole

As described in the manuscript, we performed transfer-matrix formalism (TMF) calculations assuming uniformly distributed dipoles over finite meshes as shown in Supplementary Figure 7a. For a Hertzian dipole immersed in an arbitrary medium, it is known that the total power radiated outward measured from a sphere of radius R has a simplified form of: [2]

$$P(R) \sim (2\alpha\beta R^{-3} + 4\alpha^2\beta R^{-2} + 2\alpha\beta(\alpha^2+\beta^2) R^{-1} + \beta(\alpha^2+\beta^2)^2)\, e^{-2\alpha R}, \tag{4}$$

where α and β are imaginary and real parts of the propagation constant of $\beta - j\alpha$. Therefore, while $P(R)$ becomes constant in a non-dispersive (α = 0) medium, terms scaling with R^{-3}, R^{-2}, and R^{-1} survive in a dispersive ($\alpha \neq 0$) medium and cause divergence for small R. The lowest (i.e. the most rapidly divergent) term of R^{-3} in the total energy makes the volume density of energy dissipation at each point proportional to R^{-6}. Accordingly, as shown in the bottom image of Supplementary Figure 7a, the non-radiative self-coupling (i.e. coupling with other meshes in the perovskite) in each mesh of TMF is calculated to be proportional to R^{-4}, as each mesh represents one-dimensional density along the depth (i.e. $\iint R^{-6}\, dx dy \sim R^{-4}$). It causes a significant divergence of the non-radiative self-coupling as shown in Supplementary Figure 7b, and makes the efficiency of (*outcoupled power*) / (*total dipole power*) converge to zero for small meshes according to the model of point-approximated dipole as shown in Supplementary Figure 7c. Because of such divergence, previously the finite outcoupling efficiency could have been only obtained by forcing the re-absorption to be zero. [3-7] On the other hand, we noticed that this physical contradiction could be resolved by taking PR of virtual photons into account. Unlike plasmon loss in metal, self-coupling with nearby emissive material generates another excited electron and there exists no net energy loss of the emissive layer if the newly excited electron decays radiatively. Therefore, by assuming a perfect recycling of non-radiatively self-coupled energy (white region in Supplementary Figure 7c), we can exclude this mode from the relative mode analysis for the dipole and the simulation becomes stabilised even for small mesh sizes as shown in Supplementary

Figure 7d. In systems such as quantum dots and organic molecules, each emissive/absorptive component is spatially separated. Then, the non-radiative self-coupling rate (~R^{-6}) can be quantified with a finite R and it is also known as Förster resonance energy transfer (FRET). On the other hand, it is more complicated to define the finite self-coupling rate in a continuous medium such as perovskite, and the point dipole approximation may not be appropriate for this purpose. Due to such complexity, we did not consider the diffusive motion of dipoles caused by subsequent non-radiative re-excitations in the nearby region in the calculation and assumed a uniform dipole distribution as discussed in the next section. The 100% recycling of the non-radiatively self-coupled dipole is rationalised by the infinite Purcell factor corresponding to the divergent mode. The possible losses occurring during this process can be included in η_{rad} as an electrical factor, rather than optical efficiency.

Supplementary Note 6

PR effect and LED efficiency for the future design of devices.

To provide more details to formulate design rules, Supplementary Figure 10 shows the changed EQE and PR effect according to the changed optical properties in PeLEDs, having a default structure of ITO (150 nm) / PVK (10 nm) / perovskite / TPBi (50 nm) / Al, where perovskite has a refractive index of $n_0(\lambda) + ik_0(\lambda)$ shown in Supplementary Figure 6. Compared to the EQEs for the default structure shown in Figure 3c, those for the smaller (n = 1.6) and larger (n = 2.8) real part refractive indices without PR converge to higher and lower values, respectively, due to the changed ray-optical limit of $1/2n^2$. However, even for n = 2.8, the local maxima of EQE were shown to be >20%, when it is assisted by strong microcavity effect in thin perovskite or PR in thick perovskites. For n = 1.6, in addition to its high EQE without PR, the EQE with PR is shown to further increase for thicker perovskite. In this device, because the refractive index is similar to that of the substrate (n = 1.5), most of the trapped photons propagate in the substrate mode rather than the waveguide mode. The thick perovskite layer screens Al and suppresses parasitic absorption; hence, the PR effect is maximised. On the other hand, Supplementary Figure 10b shows that a large imaginary part (k) of the refractive index is always beneficial in increasing the PR effect, despite the reduced EQE without PR. Compared to Figure 3c, the maximum EQE at 160 nm was enhanced from 20.2% to 25.1% with four times increased k, due to the enhanced PR contribution.

The PR effect is limited by the ratio between absorptions of perovskite and parasitic layers. While the non-perfect reflectivity of the metal layer is difficult to avoid, the parasitic absorption of transparent conductive electrode (TCE), which is ITO in our device, can be controlled by changing device geometry. Supplementary Figure 10c shows that the EQE with PR sharply increases as the ITO becomes thinner. Especially, when ITO is removed and no parasitic layer exists at the front side, although this may not be realistic, the maximum EQE was enhanced to 32.2 % at a perovskite thickness of 160 nm, and further increased to 34.3 % at 450 nm. In practical devices, there exists a trade-off between the transmission and conductivity of TCEs. Therefore, the optimal thickness of ITO

needs to be found to minimise both parasitic absorption loss and electrical resistivity, considering not only the direct transmission, but also the recursive PR efficiency. Adoption of novel nanomaterials such as conductive metal oxides, metal grids, metal nanowires, and graphene can be also an alternative approach to reduce the parasitic absorption loss and maximise the PR effect.

As an upper-limit of the present PeLED architecture, the best properties of Fig. S10a-c were combined and its EQE was calculated as shown in Supplementary Figure 10d. The maximum EQE of a PeLED having reduced n of 1.6 and four-times enhanced k without ITO reached 49.3% at 500 nm thickness, integrating the positive effects of the increased ray-optical limit, the metal screening effect, increased re-absorption coefficients, and reduced parasitic absorption.

Supplementary References

1. Szabo, Z. Closed form Kramers-Kronig relations to extract the refractive index of metamaterials. *IEEE Trans. Microw. Theory Tech.* **65**, 1150-1159 (2017)
2. Tai, C. T. & Collin, R. E. Radiation of a Hertzian dipole immersed in a dissipative medium. *IEEE Trans. Antennas Propag.* **48**, 1501-1506 (2000).
3. Cao, Y. *et al.* Perovskite light-emitting diodes based on spontaneously formed submicrometre-scale structures. *Nature* **562**, 249-253 (2018).
4. Zhao, B. *et al.* High-efficiency perovskite-polymer bulk heterostructure light-emitting diodes. *Nat. Photonics* **12**, 783-789 (2018).
5. Mitsas, C. L. & Siapkas, D. I. Generalized matrix-method for analysis of coherent and incoherent reflectance and transmittance of multilayer structures with rough surfaces, interfaces, and finite substrates. *Appl. Opt.* **34**, 1678-1683 (1995).
6. Benisty, H., Stanley, R. & Mayer, M. Method of source terms for dipole emission modification in modes of arbitrary planar structures. *J. Opt. Soc. Am. A* **15**, 1192-1201 (1998).
7. Lamboll, R. D. *Two-dimensional modelling of novel back-contact solar cells* Ph.D. Thesis, University of Cambridge (2017).
8. Lin, K. B. *et al.* Perovskite light-emitting diodes with external quantum efficiency exceeding 20 per cent. *Nature* **562**, 245-248 (2018).
